# Inhibition LC3B can increase chemosensitivity of ovarian cancer cells

**DOI:** 10.1186/s12935-019-0921-z

**Published:** 2019-07-29

**Authors:** Jing Tang, Jiang Zhu, Yuguang Ye, Yu Liu, Yan He, Lei Zhang, Dai Tang, Cong Qiao, Xinxin Feng, Junyi Li, Yanni Kan, Xiaobo Li, Xiaoming Jin, Dan Kong

**Affiliations:** 1 Department of Bioinformatics, Southern Medical University, Guangzhou, 510515 China; 2 Department of Pathology, Harbin Medical University, No. 157 Baojian Road, Nangang District, Harbin, 150081 China; 3 Department of Orthopedics, First Affiliated Hospital of Harbin Medical University, Harbin, 150001 China; 4 Department of Gynecology, Harbin Medical University Cancer Hospital, No. 150 Haping Road, Nangang District, Harbin, 150081 China

**Keywords:** LC3B, miR-204, Ovarian cancer, Autophagy, Drug resistance

## Abstract

**Background:**

Ovarian cancer is often accompanied by the production of ascites, and patients with repeated ascites are associated with chemotherapy resistance. The previous study confirmed that the ovarian cancer patients who developed ascites after chemotherapy had elevated autophagy levels in the ascites and precipitated cells, which was positively correlated with MDR1 expression in the blood of patients.

**Methods:**

In order to explore the correlation between autophagy and chemoresistant, we searched TCGA and GEO database to analyze the correlation between LC3B and MDR1, and identified the targeting miRNA of LC3B. It was verified by dual luciferase that miR-204 can target LC3B. The ovarian cancer cell line and the BALB/c nude mice tumor-bearing model were selected for in vitro and in vivo verification. In vitro studies confirmed that ovarian cancer cells were more sensitive to cisplatin by inhibiting LC3B.

**Results:**

Overexpression of miR-204 reduced the expression of LC3B, Atg7, and MDR1, and promoted apoptosis. In vivo studies have also confirmed that reducing the level of autophagy in ovarian cancer cells increases the sensitivity to cisplatin.

**Conclusions:**

It suggests that miR-204 can be used as a tumor suppressor gene and LC3B expression level can be used as a potential molecular marker to guide the diagnosis and treatment of patients with ovarian cancer.

**Electronic supplementary material:**

The online version of this article (10.1186/s12935-019-0921-z) contains supplementary material, which is available to authorized users.

## Background

Due to lack early symptoms and effective diagnostic methods, most patients of ovarian cancer are at an advanced stage at the time of diagnosis [1]. Conventional ovarian cancer treatment is surgery followed by chemotherapy, chemotherapy drugs are mainly platinum and adjuvant paclitaxel treatment [2]. Although ovarian cancer is a platinum-sensitive disease, about 60% of patients are resistant to cisplatin and cause recurrence of the disease, often accompanied by repeated ascites [3, 4]. Our team’s previous study found that [5], increased autophagy of tumor cells in the ascites of ovarian cancer can inhibit apoptosis, and the resistance of patients with repeated ascites may be related to the level of autophagy.

Autophagy plays a double-edged sword in the development of tumors. In normal tissues, autophagy-mediated damage repair mechanism can inhibit tumor formation, while in tumor cells, autophagy-mediated macromolecular recycling can resist external pressure, provide energy and raw materials for the survival of the tumor [6, 7]. When tumor cells are stimulated by chemotherapeutic drugs, autophagy signaling pathway is activated, autophagy level is increased, and cell survival is promoted [8]. Wang et al. found that ERK/MAPK signaling can enhance autophagy and enhance ovarian cancer cell resistance to cisplatin [9]. On the contrary, inhibition of autophagy can increase the sensitivity of tumor cells to chemotherapeutic drugs [10].

MAP1LC3B (Microtubule-associated proteins 1A/1B light chain 3B, LC3B) is the coding gene of LC3, which is the central protein in the autophagy pathway [11]. During autophagy, the carboxy terminus of LC3 is rapidly cleaved to produce cytoplasmic LC3-I [12]. LC3-I is lipidated to LC3-II, thereby binding LC3 to autophagic vesicles. The presence of LC3 in autophagosomes, as well as LC3-II, which is transformed into a downward migration, is a hallmark of autophagy [13]. Thus intracellular autophagy changes can be monitored by cell distribution and changes in LC3B. In this study, we hypothesize that LC3B might affect the sensitivity of ovarian cancer cells to cisplatin and thereby determine the prognosis of ovarian cancer. To verify the hypothesis, we analyzed the association between LC3B and MDR1, and searched the target miRNA of LC3B, assessed the effect of LC3B, miRNA and platinum chemosensitivity, and explored the possible molecular mechanism. This study provides an essential basis to identify novel treatment targets and improve chemosensitivity in ovarian cancer.

## Materials and methods

### Collection and processing of patient samples

116 cases of malignant ascites were obtained from the Third Affiliated Hospital of Harbin Medical University (Harbin, China) between January 2014 and May 2018. Of the 116 cases of ovarian cancer ascites, 64 cases were treated without chemotherapy, and 52 cases were treated with chemotherapy. We also acquired 60 samples of fresh ovarian cancerous tissue. Of the 60 cases of ovarian cancer tissue, 14 cases were treated without chemotherapy, and 46 cases were treated with chemotherapy. All cases had complete clinical and pathological data (Additional file 1: Table S1). According to the screening criteria for *Ovarian cancer, version 3.2012*. published by NCCN [14]. The patients who underwent chemotherapy were further divided into the chemosensitive (sensitive) group and the chemoresistant (resistant) group. A flow diagram describing the study and groups is shown in Additional file 2: Fig. S1 and Additional file 1: Table S2.

The supernatants of ovarian cancer ascites was stored at − 80 °C for subsequent analysis by ELISA. Then, moderate ACK lysis (Leagene Biotechnology, China) buffer was added to the precipitates, which were collected into 1.5-mL centrifuge tubes. Subsequently, RNA and protein analyzes were performed.

### Ovarian carcinoma cell lines and culture

Four ovarian cancer cell lines (OVCAR3, SKOV3, A2780 and HO8910) and human embryonic kidney cell line (HEK-293TN) were obtained from the Cell Bank, China Academy of Sciences (Shanghai, China). The cells were maintained in DMEM, MycCoy’ 5A or RIPM1640 complete medium supplemented with 2 mM glutamine and 10% fetal bovine serum (FBS) at 37 °C in a humidified atmosphere containing 5% CO_2_. Through a cytotoxicity assay (Additional file 3: Fig. S2A), we found that A2780 (IC50 = 30 μM) and HO8910 (IC50 = 37.5 μM) cells were more sensitive to cisplatin and SKOV3 (IC50 = 45 μM) and OVCAR3 (IC50 = 45 μM) cells were less sensitive to cisplatin. Accordingly, for follow-up experiments, we considered A2780 and HO8910 cells to be cisplatin high sensitivity. OVCAR3 and SKOV3 cells to be cisplatin low sensitivity.

### Establishment of ovarian cancer animal models

Twenty female BALB/c nude mice, 6–8 weeks old, were purchased from Vital River Laboratory Animal Technology Co., Ltd. (Beijing, China) and were reared under aseptic conditions. Moreover, 5 × 10^6^ OVCAR3 cells stably expressing a luciferase reporter gene, were injected into the abdominal cavity of female BALB/c nude mice. Tumor formation was observed daily and recorded using the Bruker live imaging system (Bruker, Karlsruhe, Germany). After the tumor formation, 20 BALB/c nude mice with ovarian cancer were randomly divided into four groups: cisplatin group, cisplatin and miR-204 agomir group (miR-204 agomir), cisplatin and 3-methyladenine (3MA) group and cisplatin and miR-negative group with each group containing five mice. Drug injection occurred every 3 days for 3 weeks. Doses of miR-204 agomir and miR-negative (Ribobio, Guangzhou, China) were 5 nmol, 3MA (Selleck, Shanghai, China) was 20 nmol and cisplatin (Selleck, Shanghai, China) was 5 mg/kg. The mice were sacrificed after 3 weeks following the start of treatment conditions (Fig. 5a). The collected tissue was subjected to H&E staining, western blotting, immunofluorescence and other experiments. All animals experiments strictly complied with the various standards found in the “Regulations for the Management of Experimental Animals” of Harbin Medical University. The ethical certification number is HMUIRB20170022.

### Ovarian cancer databases and data processing

We utilized data from TCGA (311 samples) and GEO databases (189 samples from GSE17260, GSE32062, and GSE32063), which explored the association between LC3B and MDR1 mRNA expression in ovarian cancer, and access the effect of LC3B on ovarian cancer overall survival (OS) and progression free survival (PFS). All TCGA data were downloaded from the GDSC Broad Institute (http://firebrowse.org). GEPIA (http://gepia.cancer-pku.cn) was used to access the effect of LC3B on pancancer OS.

### Candidate miRNA prediction

Candidate miRNAs targeting LC3B were predicted using the TargetScan algorithm (http://www.targetscan.org/) and miRbase (http://www.mirbase.org).

### Quantitative real-time polymerase chain reaction (qRT-PCR)

The expression level of miR-204 was measured in EOC samples and cells by qRT-PCR to determine the clinical implications of miR-204 expression in ovarian cancer. We also performed qRT-PCR to compare the expression of autophagy, apoptosis and resistance markers, including LC3B, Beclin1, caspase 3, caspase 9 and MDR1, in miR-204 mimic/inhibitor-transfected and negative control miR (miR-negative)-transfected cells. Total RNA was extracted from fresh tissues and cell lines using TRIzol reagent (Invitrogen, USA). Stem-loop qRT-PCR for mature miRNAs was performed using a LC96 Real-Time PCR Detection System. All PCR reactions were performed in triplicate and normalized using the comparative threshold method (2^−∆∆Ct^). The sequences of primers used for quantitative PCR, which were synthesized by GENEWIZ, are listed in Additional file 1: Table S3.

### Transfection of the miR-204 mimic/inhibitor

A chemically modified RNA-based miRNA precursor, the miR-204 mimic/inhibitor, was purchased from Ribobio (Guangzhou, China). To transfect cells, 60 pmol of the miR-204 mimic/inhibitor was diluted in 250 ml serum-free RPIM-1640 medium with 5 ml Lipofectamine 3000 (Invitrogen, Carlsbad, CA). MiR-negative was used as a control. To validate the efficiency of transfection, miRNA expression was examined by qRT-PCR.

### Luciferase assay

The 3′-untranslated region (UTR) of LC3B was PCR amplified from genomic DNA of HEK-293TN cells. Putative binding sites for miR-204 within LC3B 3′-UTR were identified using the TargetScan algorithm (targetscan.org). Wild-type or mutated binding sites were cloned separately into the Nhel and Xhol sites of the pGL3-control vector (Promega, USA). The pGL3-control (100 ng) and pRL-TK plasmids (5 ng; for normalization) were transfected into HEK-293TN cells seeded in 24-well plates (3 × 10^4^ cells/well). The synthetic miR-204 mimic was added to the above reactions at a concentration of 20–60 nM. Luciferase activity was measured after 48 h using an Infinite 200pro series luminometer (Tecan Group, Switzerland) with the Dual-Luciferase reporter assay system (Promega, USA) according to the manufacturer’s instructions. All experiments were performed in triplicate and normalized to Renilla luciferase activity.

### Cell viability assay

Cell viability was assessed after adding different concentrations of cisplatin to cells according to the Cell Counting Kit-8 (CCK-8) assay kit (Dojindo, Japan) protocol. Absorbance was measured at 450 nm using a microplate reader.

### Enzyme-linked immunosorbent assay (ELISA)

The abundance of the LC3 protein in ovarian cancer ascites was measured by the ELISA using kits purchased from Elabscience (Wuhan, China).

### Western blot

Total protein from cells and ovarian cancer samples was extracted, and the concentration in the supernatant was measured using the Bradford assay (Sigma, USA). Equal amounts of total protein were separated by 15% sodium dodecy1 sulfate–polyacrylamide gel electrophoresis (SDS-PAGE) and transferred to nitrocellulose membranes (Millipore Co. Bedford, MA, USA). After blocking with 5% skim milk for 1 h at room temperature. The membranes were incubated overnight at 4 °C with primary antibodies (Additional file 1: Table S4). The membranes were then incubated with a horseradish peroxidase (HRP)-conjugated anti-mouse or anti-rabbit secondary antibody (1:5000, Novus Biologicals) for 1 h at room temperature. Next, the bands were visualized using an enhanced luminol-based chemiluminescence detection kit (Bio-Rad Laboratories, Hercules, CA, USA). The protein levels were normalized to GAPDH as an internal control.

### Transmission electron microscopy (TEM)

OVCAR3 cells in agarose were treated with 1% OsO4 solution for 1 h at 4 °C; this helped to provide an enhanced contrast for TEM images. Samples were dehydrated in a graded series of acetone, and then embedded in Epon 812. The samples were cut into ultrathin sections (50–70 nm in thickness), double-stained with uranyl acetate and lead citrate and examined with an electron microscope (H-7650, Hitachi, Japan). Images were transferred to Adobe Photoshop software for final processing.

### Immunofluorescence staining

The cells were fixed with 4% paraformaldehyde (PFA) for 30 min. The cells were then incubated overnight with corresponding primary antibodies. Next, the cells were incubated with secondary antibodies for 1 h at 37 °C. The cells were washed 3 times and incubated with 4′,6-diamidino-2-phenylindole (DAPI) for 5 min to stain the nuclei. Images were then obtained using a Nikon E800 Multifunctional Biological Microscope and Image Acquisition Software Nikon ACT 21 version 6.1.

### TUNEL staining

Apoptosis was determined by the terminal deoxyribonucleotide transferase-mediated (TUNEL) assay using a kit from Roche (Hamburg, Germany). Cells grown in 24-well culture clusters were treated with different concentrations of cisplatin for 24 h. The treated cells were fixed onto poly-(l-lysine)-coated slides with 4% PFA. The cells were incubated in 50 µl of TUNEL reaction mixture for 1 h at 37 °C in the dark. Next, 50 µl of DAPI was added and incubated for 5 min at room temperature. The cells were imaged using fluorescent microscopy. Cells exhibiting green fluorescence were defined as TUNEL-positive apoptosis cells.

### Transwell migration assay

Transwell chambers with 8-μm pores were obtained from Corning (Corning, USA). Transfected cells were harvested, resuspended in RPMI-1640 without FBS at a concentration of 1 × 10^5^ cells in 100 μl and then seeded into the upper chambers of a 24-well plate. The lower chambers were filled with 600 μl RPMI-1640 containing 10% FBS. The cells were incubated for 24 h. At the end of the experiment, cells that had migrated to the reverse side of the Transwell membrane were fixed with methanol, stained with crystal violet, and counted under a light microscope at 100× magnifications. An average of 10 visual fields was examined.

### Wound-healing assay

A wound-healing assay was used to assess cell migration. The two groups of cells transfected with miR-204 mimic/inhibitor or miR-negative were cultured to confluence (> 90%) in 6-well plates. A straight scratch wound was then generated with a 200-ml sterile pipette tip. After 24 h, the cells were photographed, and those migrating from the scratch line were counted. This assay was repeated three times.

### H&E staining

Tumor tissues of BALB/c nude mice were routinely processed into paraffin blocks and then sectioned at a thickness of 4 to 6 μm. The sections were then stained with H&E.

### Statistical analysis

All data were obtained based on a minimum of three experiments and were presented as the mean ± standard deviation (SD). Statistical analysis was performed using SPSS software version 21.0. One-way analysis of variance (ANOVA) was used to analyze differences between groups. An independent *t*-test or *u*-test for differences in mean values was used for comparison. Comparison between two or multiple rates was performed using the Chi square test. Differences with a *p* value of < 0.05 were considered significant. Spearman correlations were used to find linear relationships between two variables. Kaplan–Meier analysis performed a survival analysis on the prognosis of ovarian cancer patients in TCGA and GEO databases. Univariate analyses with Cox regression were used to determine the proportional hazards. The difference was statistically significant. GraphPad Prism was used for mapping and curve fitting.

## Results

### Ovarian cancer resistance is associated with increased levels of autophagy

**Fig. 1:**
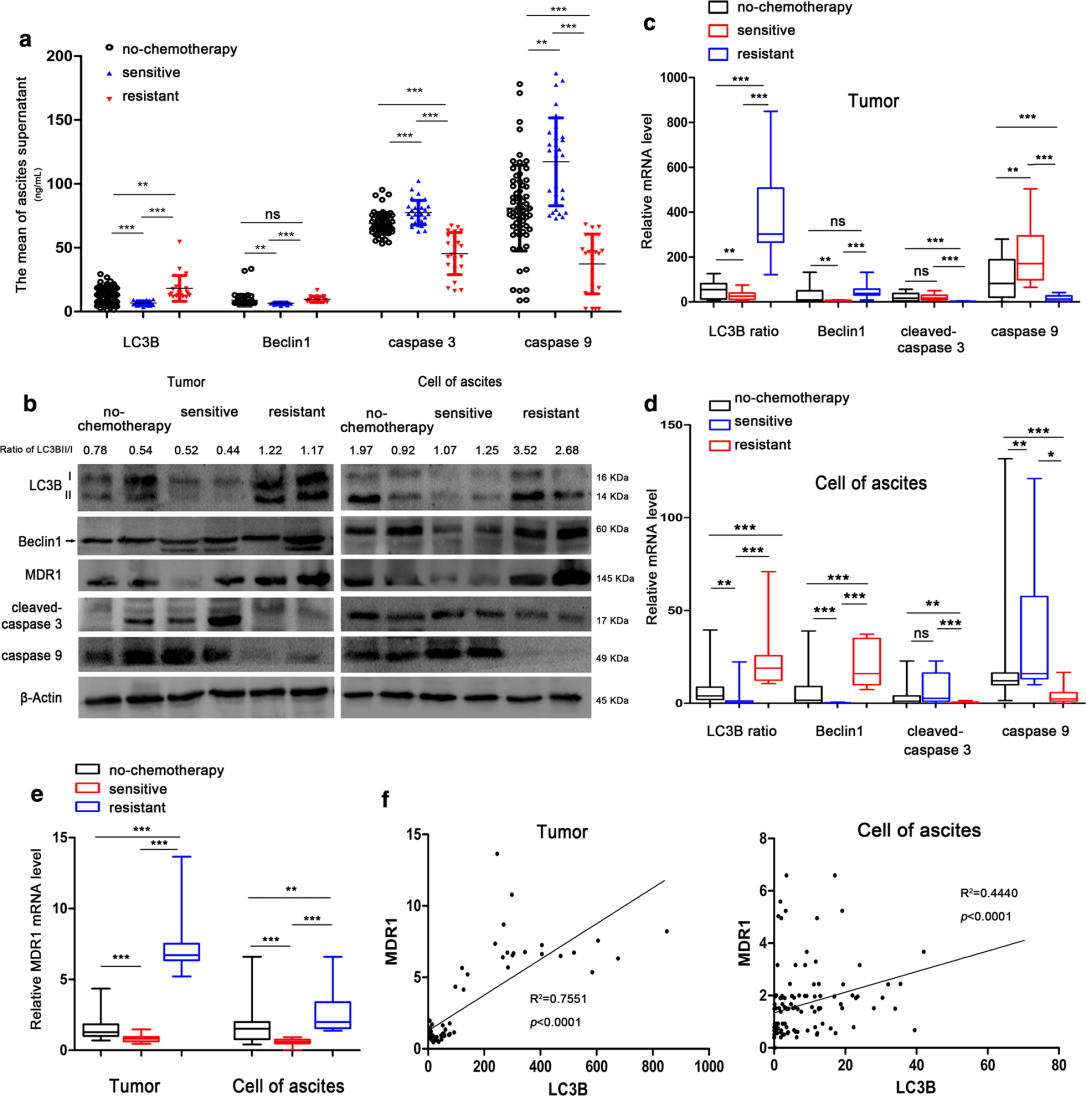
LC3B is upregulated in a chemoresistant group of EOC. **a** The LC3, Beclin1, caspase 3 and caspase 9 level in EOC ascites were measured by ELISA. n = 116 (no chemotherapy group: n = 64, sensitive group: n = 30 and resistant group: n = 22), *p < 0.05, **p < 0.01. **b** The level of LC3B, Beclin1, MDR1, cleaved-caspase 3 and caspase 9 expression in the EOC samples was measured by western blotting assay. **c**, **d** The level of LC3B, Beclin1, caspase 3 and caspase 9 expression in the EOC samples was measured by qRT-PCR assay. *p < 0.05, **p < 0.01, ***p < 0.001. **e** The MDR1 expression level in EOC samples was measured by qRT-PCR assay. *p < 0.05, **p < 0.01, ***p < 0.001. **f** The correlation between MDR1 level and LC3B in EOC tumor tissues/ascites cells was determined using Spearman correlation coefficients

The levels of autophagy and apoptosis markers in 116 cases of ovarian cancer ascites were detected by ELISA. Compared with the non-chemotherapy group and the chemosensitive (sensitive) group, the concentrations of LC3B and Beclin1 in the chemoresistant (resistant) group were increased, and the concentrations of caspase 3 and 9 were decreased (Fig. 1a). The results of qRT-PCR and western blot showed that compared with the sensitive group, the levels of LC3B and Beclin1 in the resistant group were significantly increased, the expression of caspase 3 and 9 was decreased, and the expression of MDR1 in the resistant group was higher than that in the sensitive group (Fig. 1b–e). When expression of autophagy markers increased, the expression of apoptosis protease decreased. Spearman correlation analysis showed LC3B and MDR1 were positively correlated (Fig. 1f, *p *< 0.0001). Correlation analysis between LC3B and MDR1 was also positively correlated in ovarian cancer samples from TCGA and GEO databases (Additional file 3: Fig. S2B, *p *< 0.005). These results were suggesting that ovarian cancer resistance may be associated with LC3B.

### Cell level verification that LC3B changes affect tumor cell resistance

**Fig. 2:**
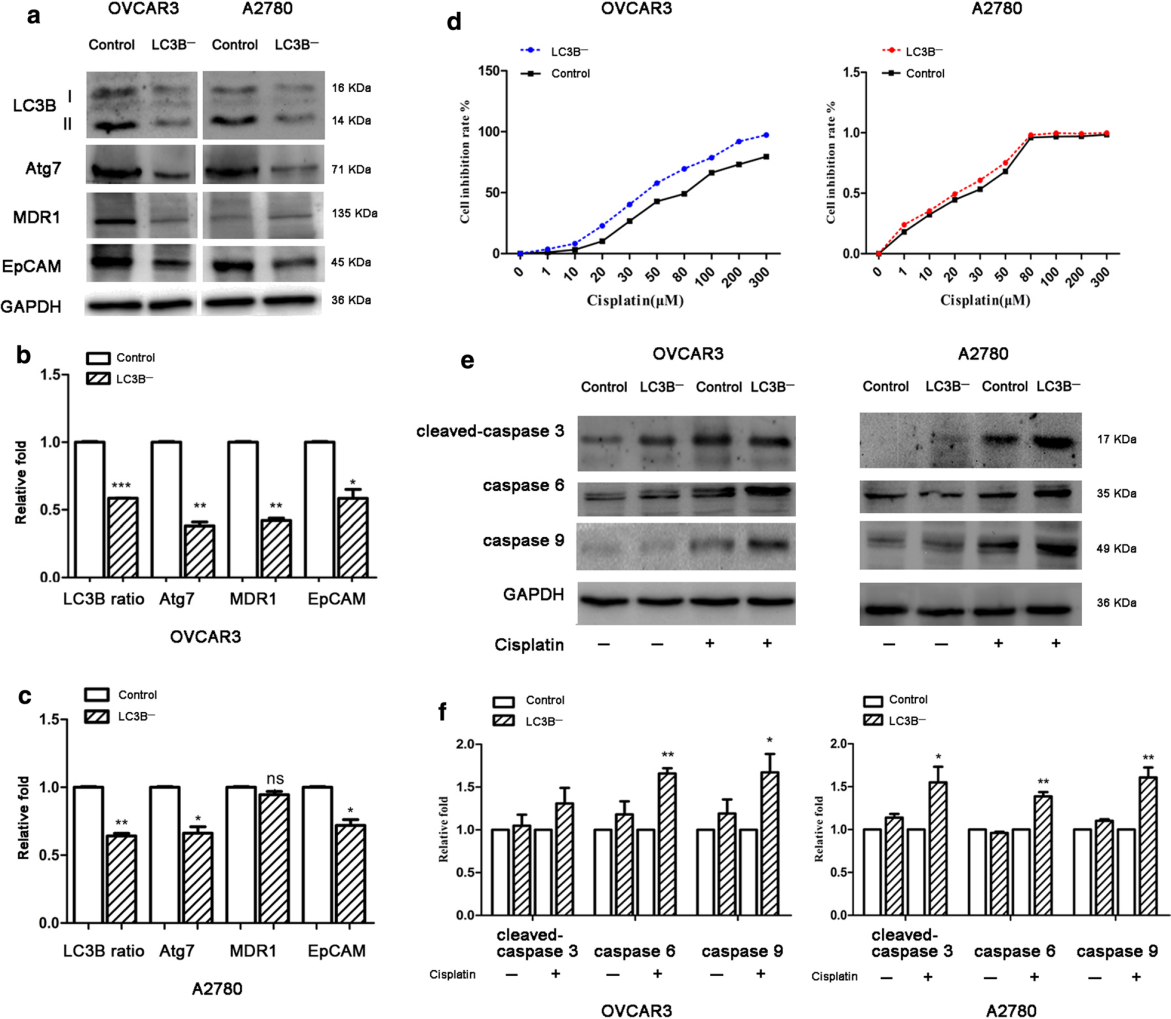
Knockdown LC3B in ovarian cancer cells enhance sensitive of tumor cell to cisplatin. **a**–**c** Western blot and statistical analysis of LC3B, Atg7, MDR1 and EpCAM expression in ovarian cancer cells. **p < 0.01, ***p < 0.001. **d** Cell viability was assessed by CCK-8 in LC3B- group and control group. **e**–**g** The level of cleaved-caspase 3, caspase 6 and caspase 9 expression in ovarian cancer cells was measured by western blotting assay and statistical analysis. *p < 0.05, **p < 0.01, ***p < 0.001

The level of LC3B expression in the EOC may affect the response of tumor cells to chemotherapeutic drugs. We identify this hypothesis through ovarian tumor cell lines. By knocking down LC3B (LC3B^−^) in ovarian cancer cell lines, western blot showed that compared with the control group, the expression of LC3B, Atg7 and MDR1 was decreased in the LC3B^−^ group (Fig. 2a–c). CCK8 results showed that cisplatin had a higher inhibition rate in the LC3B^−^ group than in the control group, and the sensitivity of the LC3B^−^ group was significantly increased (Fig. 2d). The western blot results showed compared with the control group, the expression of apoptosis proteins caspase 3, 6, and 9 in the LC3B^−^ group was significantly increased (Fig. 2e, f). These results suggested that LC3B expression could affect the sensitivity of tumor cells to cisplatin. The expression of LC3B was decreased. The tumor cells were more sensitive to cisplatin.

### miR-204 targeting LC3B inhibits autophagy

The LC3B expression could effect on ovarian cancer resistance, but the causes leading to LC3B expression change in ovarian cancer were unknown. So we searched for computationally predicted candidate miRNAs using a web-based miRNA database (http://www.targetscan.org). Among multiple candidate miRNAs with high potential binding capacity to the 3′-UTR of LC3B, four putative miRNAs (miR-204, miR-211 and miR-182, miR-212) were selected. Four miRNA levels were detected in ovarian cancer samples (ascites and tissues). The expression of miR-204 in the resistant group was significantly decreased in both tumor tissues and ascites cells (Additional file 3: Fig. S2C). It was speculated that LC3B expression may be related to miR-204 deletion in ovarian cancer. At the same time, the expression level of four miRNAs was also detected in ovarian cancer cell lines. The expression of miR-204 in A2780 was significantly higher than that in OVCAR3 (Additional file 3: Fig. S2D). Therefore, it was used miR-204 for subsequent experiments. The results showed that OVCAR3 was less sensitive to cisplatin and A2780 was more sensitive (Additional file 3: Fig. S2A). So subsequent in vitro validation was performed using OVCAR3 and A2780 cell lines.

**Fig. 3:**
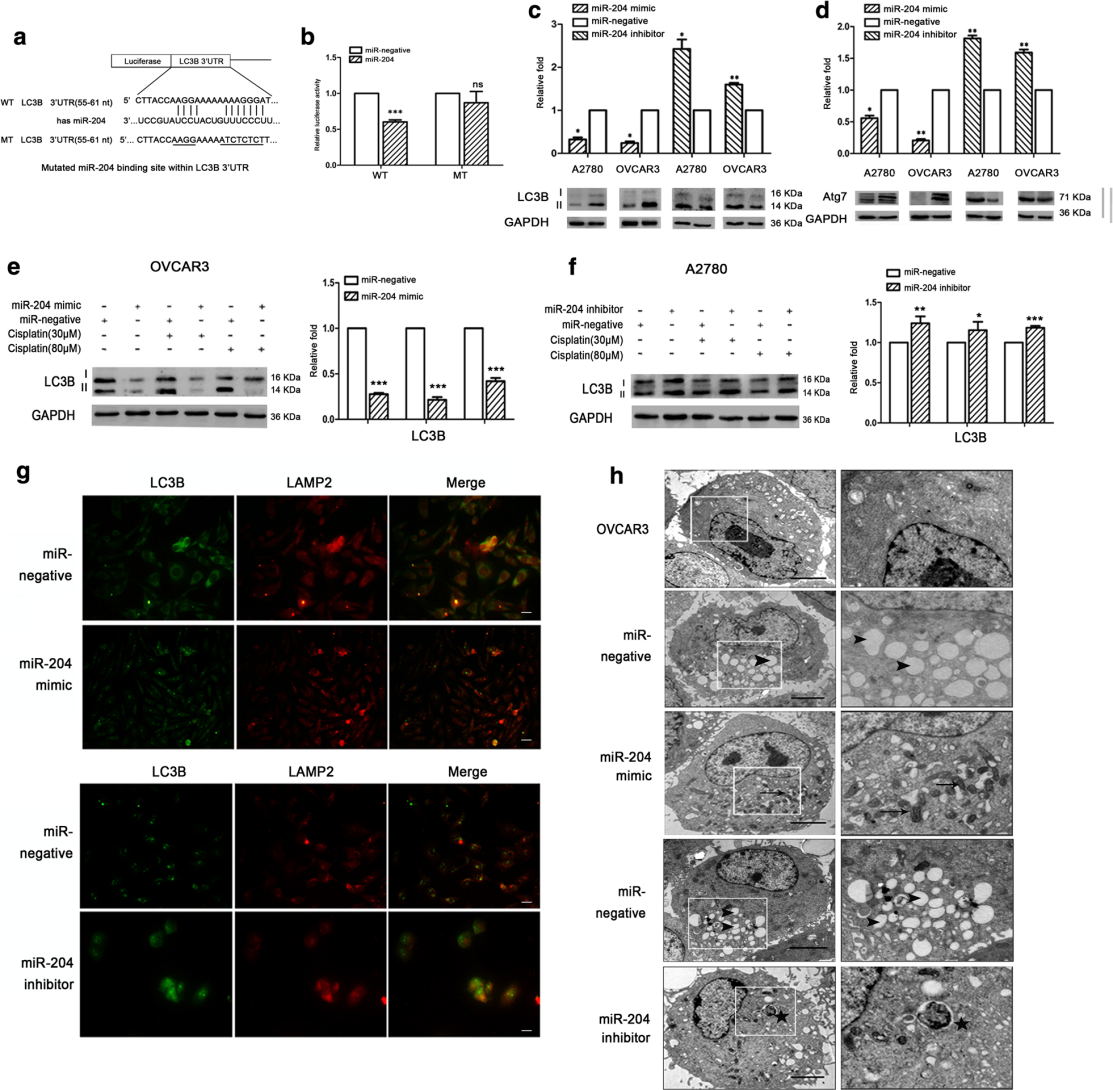
Validation of candidate miRNAs targeting LC3B in EOC. **a** Schematic diagram indicating the wild-type and mutated binding sites of miR-204 in the LC3B 3′-UTR. **b** Co-transfection of miR-204 reduced LC3B luciferase activity in HEK-293TN cells compared with the miR-negative control, whereas this miRNA had no effect on the mutant LC3B 3′-UTR, ***p < 0.001. **c**, **d** Western blot and statistical analysis of LC3B and Atg7 expression in OVCAR3 and A2780. LC3B and Atg7 expression were normalized to that of GAPDH. *p < 0.05, **p < 0.01. **e**, **f** Western blotting. The LC3B protein in OVCAR3/A2780 cells after transfection. In the presence of 30 μM and 80 μM cisplatin, expression of LC3B was significantly decreased/increased in the miR-204 mimic/inhibitor group compared to the miR-negative group. Protein expression was normalized to that of GAPDH. *p < 0.05, **p < 0.01, ***p < 0.001. **g** Immunofluorescence staining. Transfection of OVCAR3/A2780 cells with miR-204 mimic/inhibitor or miR-negative. LC3B staining was green, LAMP2 staining was red. Scale bars: 10 µm. **h** Electron microscopy. Organelle changes in OVCAR3 cells after transfection of miR-204 mimic, inhibitor or miR-negative. The scale bar represents 2 μm. The arrow represents mitochondrion, the triangle-arrow represents lipid droplets, and the star represents autophagic vacuoles

Luciferase assay were performed to whether miR-204 directly targets the LC3B mRNA. The binding sites of miR-204 and LC3B are shown in Fig. 3a. Intact and mutated 3′UTRs of LC3B, were cloned into a luciferase reporter plasmid that was co-transfected with a miR-204 mimic into HEK 293TN cells. miR-204 reduced the luciferase activity of the 3′-UTR reporter of wild type LC3B, while that of the mutated reporter was not significantly affected (Fig. 3b and Additional file 1: Table S5). Transfection with the miR-204 mimic significantly downregulated LC3B (Fig. 3c, *p *< 0.001) and Atg7 (Fig. 3d, *p *< 0.001) expression in OVCAR3 cells and A2780 cells. Treatment of ovarian cancer cells with cisplatin showed a significant decrease expression of LC3B in the miR-204 mimic group (Fig. 3e) and increased expression of LC3B in the miR-204 inhibitor group (Fig. 3f). Immunofluorescent staining showed the co-localization of LC3B and LAMP2 in the miR-204 mimic group was significantly reduced, and the co-localization of LC3B and LAMP2 in the miR-204 inhibitor group was increased (Fig. 3g). Electron microscopy showed that OVCAR3 cells have large nuclei and abundant nucleosomes. In the miR-negative group, more lipid droplets were seen in the cytoplasm. In the miR-204 mimic group, there were large numbers of proliferating mitochondria. In the miR-204 inhibitor group, there were large number of autophagic lysosomes were seen in the cytoplasm (Fig. 3h). These data indicated that miR-204 directly targets the 3′-UTR of LC3B. It repressed the expression of the LC3B protein, and miR-204 could inhibit autophagy by targeting LC3B.

### miR-204 inhibits LC3B and promotes apoptosis

**Fig. 4:**
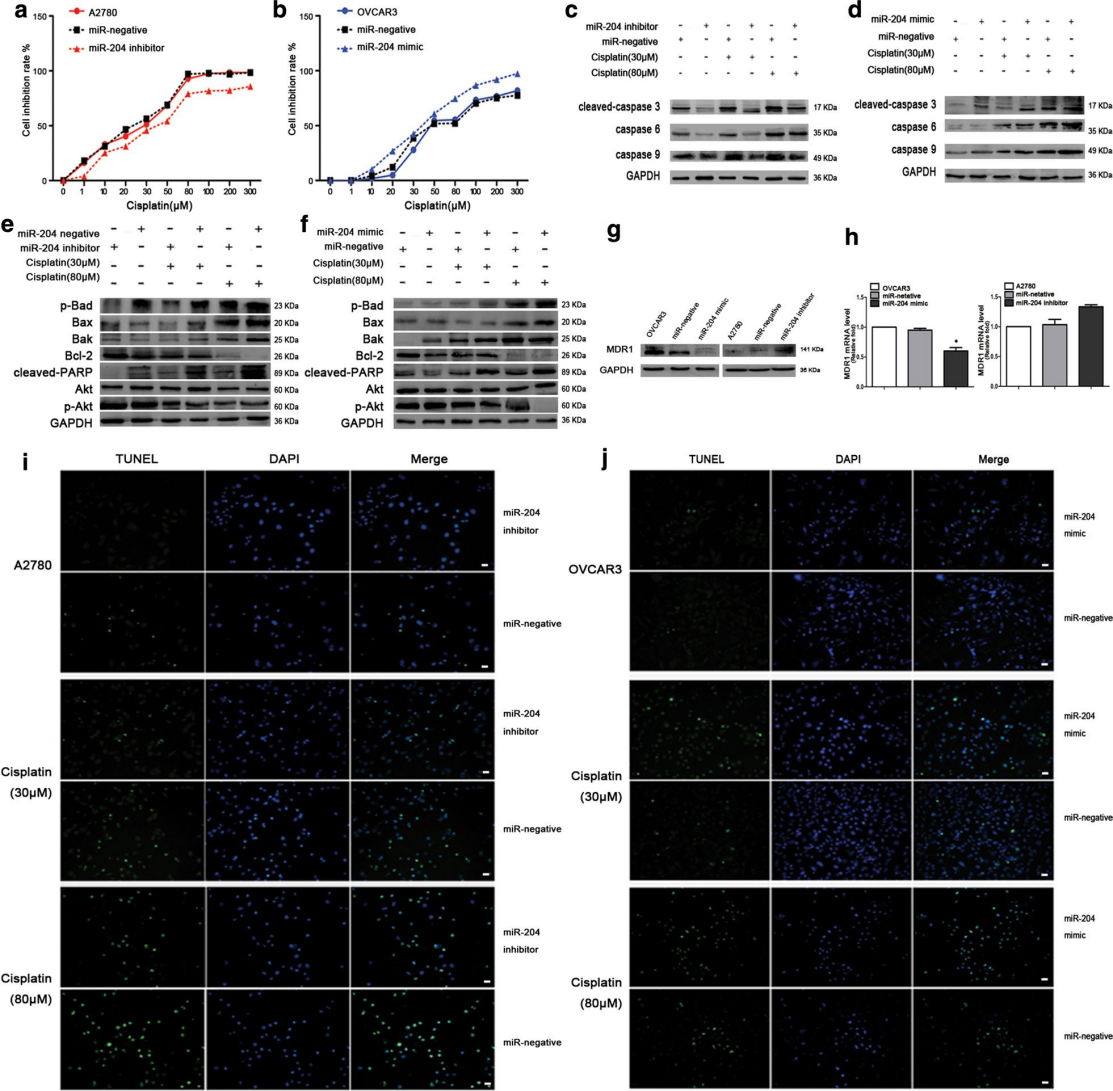
miR-204 enhances cisplatin-induced cytotoxicity in the ovarian cancer cells. **a**, **b** Cell viability was assessed by CCK-8 after treatment of A2780 and OVCAR3 cells with cisplatin alone or in combination with miR-204 inhibitor/mimic or miR-negative transfection. **c**, **d** Western blotting. Transfection with the miR-204 inhibitor/mimic increased/decreased cleaved-caspase 3, caspase 6 and caspase 9 expression in A2780/OVCAR3 cells. Protein expression was normalized to that of GAPDH. **e**, **f** Western blotting. Transfection with the miR-204 inhibitor/mimic increased/decreased p-Bad, Bax, Bak, Bcl-2, cleaved-PARP, Akt and p-Akt expression in A2780/OVCAR3 cells. Protein expression was normalized to that of GAPDH. **g** Western blotting. Transfection with the miR-204 mimic/inhibitor decreased/increased MDR1 expression in OVCAR3/A2780 cells. MDR1 expression was normalized to that of GAPDH. **h** qRT-PCR assay. Transfection with the miR-204 mimic/inhibitor decreased/increased MDR1 mRNA expression in OVCAR3/A2780 cells, *p < 0.05. **i**, **j** TUNEL staining. Transfection of A2780/OVCAR3 cells with the miR-204 inhibitor/mimic decreased/increased apoptosis compared with the miR-negative group. Apoptosis cells (green), DAPI-stained nuclei (blue). Scale bars: 10 µm

The results of ovarian cancer samples (tissues and ascites) showed apoptosis protease was decreased when LC3B expression was increased in the resistant group, while the apoptosis of ovarian cancer cells was increased when LC3B was inhibited. Due to miR-204 was directly targeted at LC3B. We hypothesized that the mechanism of LC3B expression decreased and apoptosis increased was related to miR-204. CCK8 results showed that the A2780 cells were less sensitive to cisplatin and increased cell viability in miR-204 inhibitor group (Fig. 4a and Additional file 4: Fig. S3A); the OVCAR3 cells were more sensitive to cisplatin and cell viability was significantly reduced in the miR-204 mimic group (Fig. 4b and Additional file 4: S3B). Western blot analysis showed that cleaved-caspase 3, caspase 6, caspase 9, p-Bad, Bax, Bak and cleaved-PARP were decreased andBcl-2, p-Akt and MDR1 were increased in miR-204 inhibitor group. Conversely, cleaved-caspase 3, caspase 6, caspase 9, p-Bad, Bax, Bak and cleaved-PARP were significantly increased and Bcl-2, p-Akt and MDR1 were decreased in miR-204 mimic group (Fig. 4c–h). Western gray value statistics are shown in Additional file 4: Fig. S3C-F. RT-PCR results showed that the mRNA level of caspase 3 and caspase 9 was increased in the miR-204 mimic group and decreased in the miR-204 inhibitor group compared with the control group (Additional file 4: Fig. S3G-H). TUNEL assay showed that a decrease in the number of apoptosis cells in the miR-204 inhibitor group compared to the control group (Fig. 4i); whereas the number of apoptosis cells in the miR-204 mimic group increased (Fig. 4j). Above all, miR-204 could inhibit LC3B and promote apoptosis at the same time in ovarian cancer cells.

### Tumor-bearing model of BALB/c nude mice demonstrates inhibition of LC3B on tumors

**Fig. 5:**
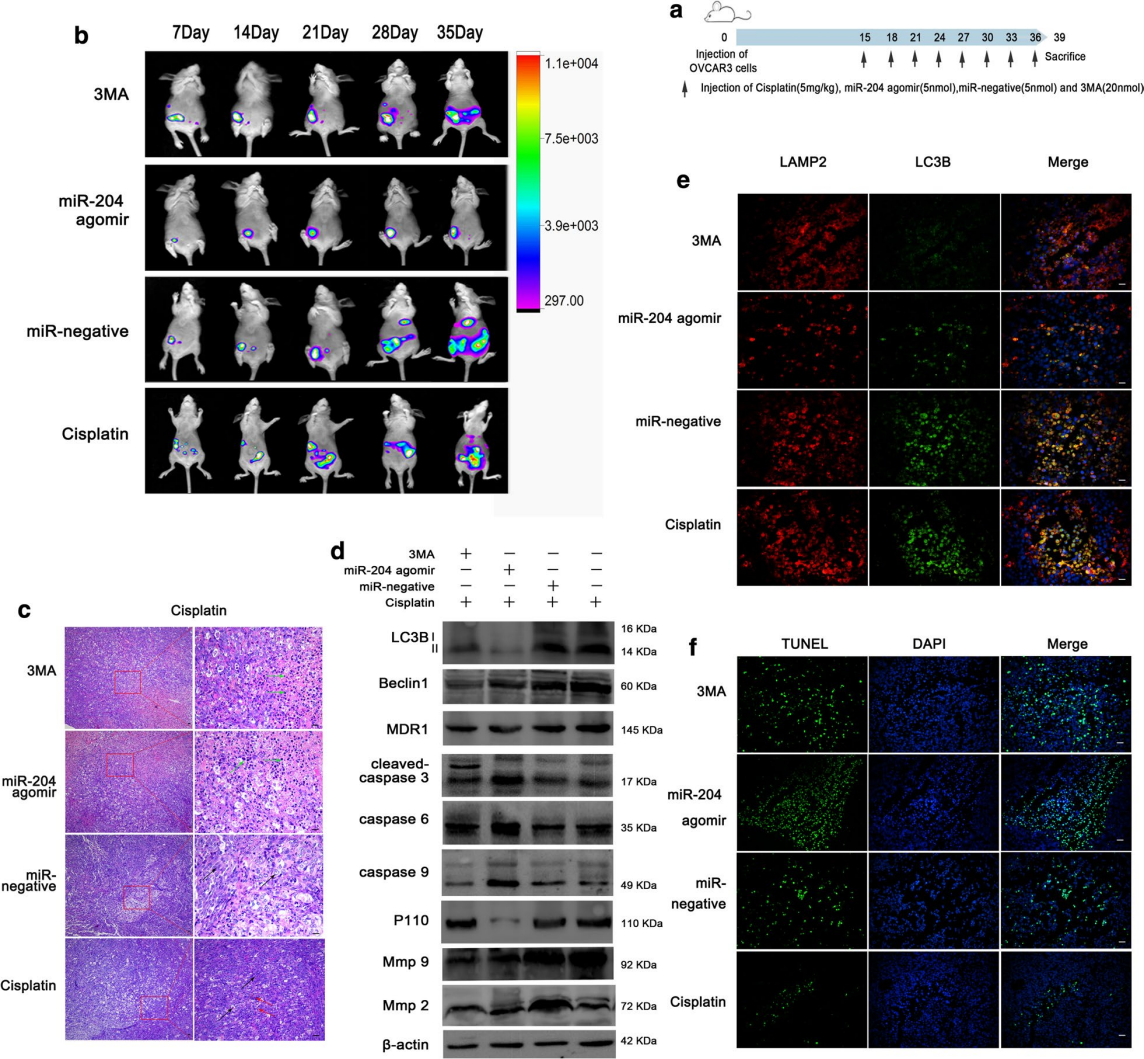
Inhibit LC3B could promote apoptosis in vivo. **a** Schematic diagram of drug injection after the establishment of an abdominal cavity model of nude mouse ovarian cancer. **b** Representative images of the Bruker imaging system tracking the tumor process in vivo. **c** H&E staining. Green arrow shows apoptotic cells, red arrow shows inflammatory cells, and black arrow shows tumor cells. Scale bars: 10 µm. **d** Western blotting and statistical analysis were used to detect the expression of LC3B, Beclin1, MDR1, cleaved-caspase 3, caspase 6, caspase 9, P110, MMP2 and MMP9 in tumor tissue. **e** Dual fluorescence of LC3B and LAMP2 detection of autophagy expression in tumor tissue, LAMP2 (red), LC3B (green), DAPI (blue), co-localization (yellow). Scale bars: 10 µm. **f** TUNEL assay. Apoptosis cell (green), DAPI (blue). Scale bars: 10 µm

In vivo experiments were performed on a nude mouse model of intraperitoneal tumors. As showed in Fig. 5b, the miR-204 agomir group had a slower tumor growth rate than the other three groups. ROI values result is shown in Additional file 3: Figure S2E. H&E staining results showed that apoptosis necrosis occurred in many tumor cells in miR-204 agomir group and 3MA group. In cisplatin alone and the control group, tumor cells showed less apoptosis and a small amount of vacuoles with inflammation (Fig. 5c). Western Blot assays showed that compared with the control group and the cisplatin group, LC3B and Beclin1 were lower in miR-204 agomir and 3MA groups, cleaved-caspase 3, 6, and 9 were increased, and MMP2 and MMP9 were decreased. Compared with the other three groups, P110 and MDR1 were significantly decreased in the miR-204 agomir group (Fig. 5d). The results of double immunofluorescence staining showed that the 3MA and miR-204 agomir groups had fewer co-localization, and the other two groups had more co-localization, indicating that autophagy was decreased in the 3MA and miR-204 agomir groups (Fig. 5e). TUNEL assay showed that compared with the other two groups, miR-204 agomir and 3MA group had increased apoptosis, and apoptosis in the miR-204 agomir group was more significant (Fig. 5f).

Above results indicate that miR-204 agomir and 3MA can inhibit tumor autophagy. When tumor autophagy was inhibited, apoptosis also changed. Compared with the 3MA group, apoptosis was more obvious in the miR-204 agomir group. It indicated that miR-204 could inhibit autophagy,promote tumor cell apoptosis and increase the sensitivity of tumor cells to cisplatin.

### LC3B expression affect ovarian cancer tumor cell migration

**Fig. 6:**
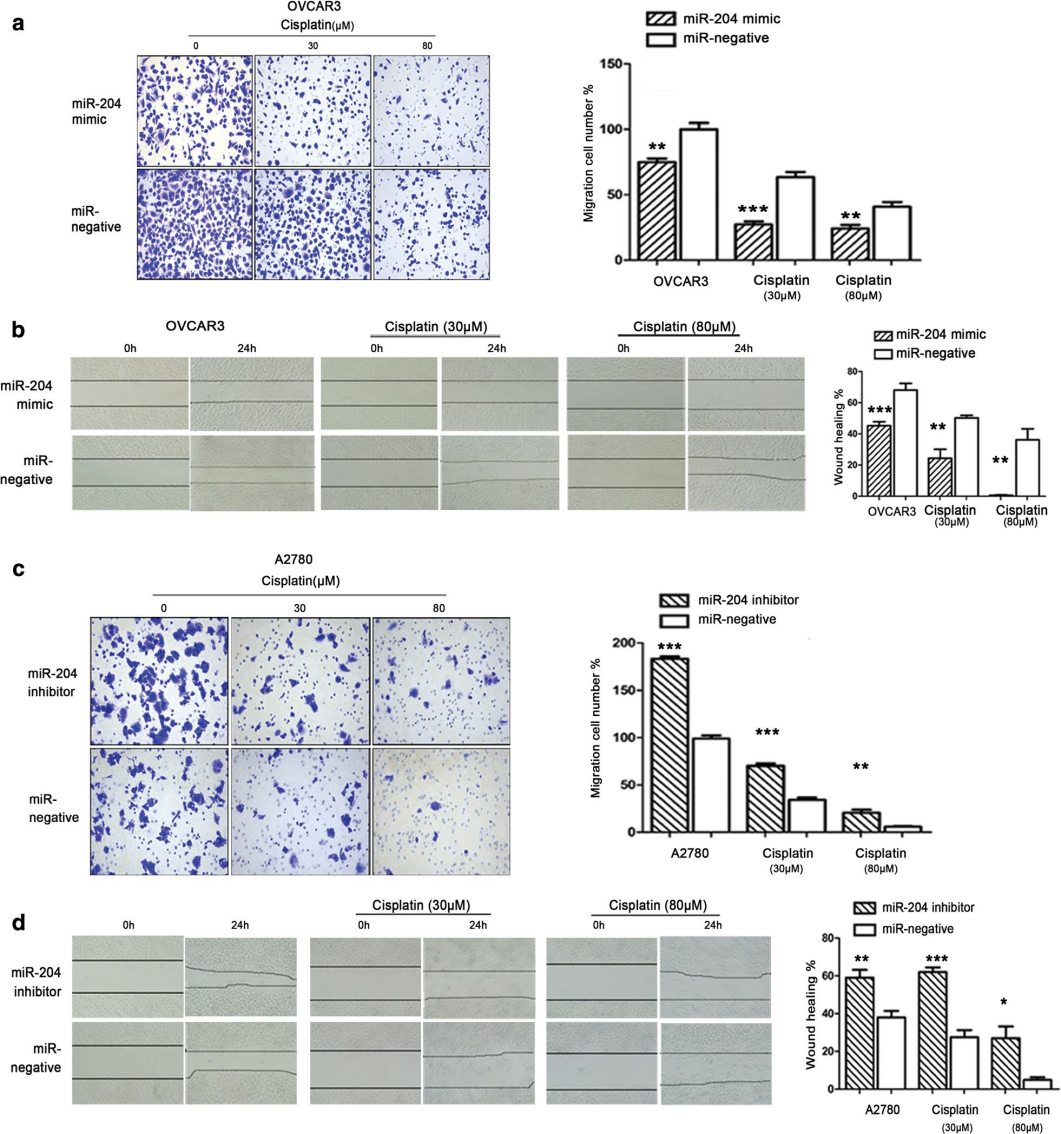
Inhibit LC3B could affect tumor cell migration. **a** OVCAR3 cells transfected with miR-204 mimic or miR-negative were subjected to Transwell assays; samples were photographed and quantified. miR-204 overexpression inhibited migration of OVCAR3 cells relative to the miR-negative group (magnification, ×200). **p < 0.01, ***p < 0.001. The graph represents the mean ± SD of triplicate experiments. **b** Representative pictures of wound-healing assays show the closing of open wounds introduced in confluently grown transfected cells after 24 h in OVCAR3 cells, **p < 0.01,***p < 0.001, magnification, ×100. **c** A2780 cells transfected with miR-204 inhibitor or miR-negative were subjected to Transwell assays; samples were photographed and quantified. miR-204 inhibitor enhanced migration of A2780 cells relative to the miR-negative group (magnification, ×200). **p < 0.01, ***p < 0.001. The graph represents the mean ± SD of triplicate experiments. **d** Representative pictures of wound-healing assays show the closing of open wounds introduced in confluently grown transfected cells after 24 h in A2780 cells, **p < 0.01,***p < 0.001, magnification, ×100

In vivo studies showed that MMP2 and MMP9 were decreased in miR-204 agomir group and 3MA group. MMP2 and MMP9 were members of metal matrix protease family, which was closely related to tumor migration. The results in vivo suggested that inhibition of autophagy could effect on tumor migration. Therefore, the effects of LC3B expression on migration and invasion in ovarian cancer cells were verified by Transwell and wound-healing experiments. Transwell assay showed that the cell migration rate of the miR-204 mimic group was decreased compared with the control group. After the addition treatment of cisplatin, the migration rate of the miR-204 mimic group was significantly decreased. While the migration rate of the miR-204 inhibitor group was increased compared to the control group, and after treatment with cisplatin, the cell migration rate was significantly increased (Fig. 6a, c). The results of wound-healing assays were consistently with transwell assays (Fig. 6b, d). These results indicated miR-204 inhibited LC3B expression could affect migration in ovarian cancer cell.

### LC3B was related to ovarian cancer prognosis

**Fig. 7:**
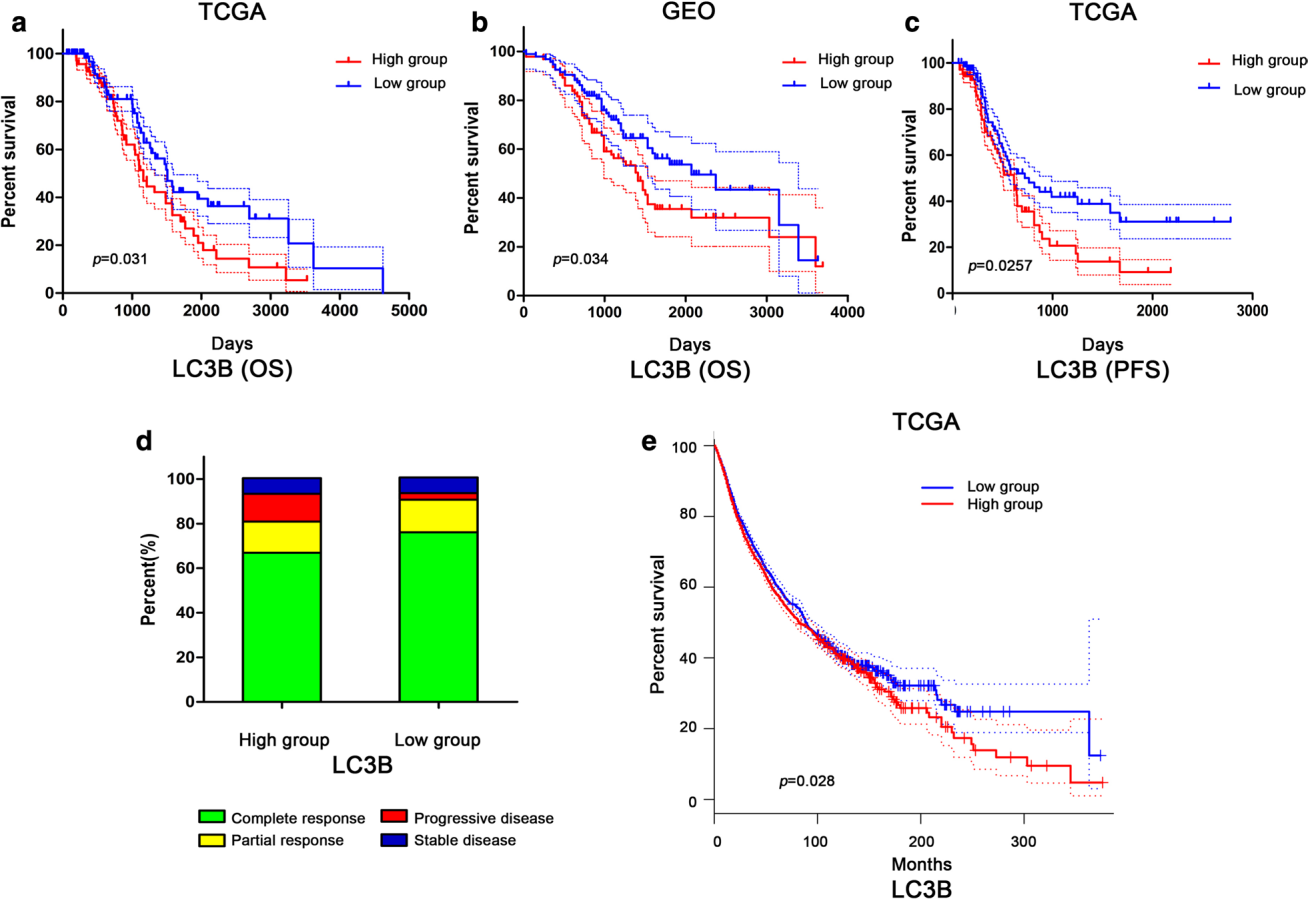
Prognostic analysis of LC3B in TCGA and GEO database. **a** Kaplan–Meier and Cox analysis of OS in TCGA data (n = 311), high group > 75% of LC3B level, low group < 25% of LC3B level. **b** Kaplan–Meier and Cox analysis of OS in GEO data (n = 189), high group > median LC3B, low grou*p *< median LC3B. **c** Kaplan–Meier and Cox analysis of PFS in TCGA data (n = 311). high group > 75% of LC3B level, low group < 25% of LC3B level. **d** Statistical analysis of complete platinum response, partial response, progressive disease and stable disease in TCGA data. **e** Prognostic analysis of pancancer samples in TCGA (n = 9502) use GEPIA. High group > median LC3B, low group < median LC3B

To identify clinical application value of LC3B, we analyzed the effect of LC3B on overall survival (OS) of ovarian cancer patients based on a set of TCGA data, including ovarian cancer patients (n = 311) receiving platinum treatment. The result indicated that patients with low levels of LC3B exhibit a significantly longer survival time compared with those with high levels of LC3B (Fig. 7a). We also performed univariate analysis using Cox proportional hazard regression. High LC3B expression was associated with a worse OS, and low expression was associated with improved OS (high vs. low by quartile, HR of death = 1.655, 95% CI 1.048–2.614, p = 0.031). At the same time, 189 ovarian cancer samples obtained from 3 GEO databases were analyzed. The effect of LC3B expression on the overall survival of patients was consistent with results of TCGA data (Fig. 7b, high vs. low by median, HR of death = 1.567, 95% CI 1.035–2.373, p = 0.034). We also analyzed the effect of LC3B on PFS of patients based on TCGA data. The results showed that patients with low LC3B expression had longer PFS than those with high LC3B expression (Fig. 7c, *p *= 0.0257). In addition, LC3B was associated with the consequence of platinum treatment in ovarian cancer patients (Fig. 7d). Compared with the high expression group, low level of LC3B (76.1% VS% 66.9%) had significantly more complete platinum response. These results indicated that LC3B could be used as a prognostic indicator of ovarian cancer, and LC3B could also play such a role in the other cancer. We used GEPIA website (http://gepia.cancer-pku.cn) to analyze multiple cancer samples in TCGA database. The results showed that the prognosis of low LC3B expression group was better in squamous cell lung cancer, colon cancer and esophageal cancer (Additional file 4: Fig. S3I). Analysis of pancancer samples in TCGA (n = 9502) showed that low LC3B expression group had a better prognosis (Fig. 7e). Above all, it was suggested that LC3B could be a biomarker for predicting the prognosis of cancer.

## Discussion

Patients with epithelial serous ovarian cancer are often associated with ascites in the advanced stage, which is one of the problems in the treatment of ovarian cancer [15]. In the previous study, our team found that ovarian cancer patients had different levels of autophagy in their ascites [5]. In resistant group of ovarian cancer patients, the autophagy was increased and apoptosis was decreased, respectively. It was speculated that resistant of ovarian cancer may be related to the increase of autophagy. We hypothesized that resistant of ovarian cancer cells were associated with LC3B expression, which was the key of autophagy genes. Ovarian cancer samples results showed that there was a positive correlation between LC3B and MDR1, and correlation analysis in ovarian cancer samples from TCGA and GEO databases also indicated a positive correlation between LC3B and MDR1. Knockdown LC3B in ovarian cancer cells, which apoptosis was increased and the MDR1 expression was decreased. Tumor model also showed the apoptosis was increased in the 3MA group and miR-204 agomir treatment group. The sensitivity of tumor cells to cisplatin was increased.

Due to further study the mechanism of LC3B affecting chemosensitivity in ovarian cancer cells, targeting miRNAs of LC3B was identified by miRbase and TargetScan. Dual luciferase assay showed that miR-204 could directly target LC3B, which was consistent with the findings of Olga Mikhaylova et al. in renal clear cell carcinoma [16]. The results of ovarian cancer samples (ascites and tissues) showed that the level of miR-204 in the resistant group was significantly lower than that in the sensitive group, miR-204 was negatively correlated with LC3B (Additional file 3: Fig. S2F).

During autophagy, the adaptor protein p62/SQSTM1 is consumed, and LC3 conversion is promoted [17]. Obstruction of autophagy flux can be induced artificially by chloroquine and bafilomycin A1, both of which result in increased levels of ubiquitination, p62 activation, and LC3-II accumulation. The smooth autophagy-lysosome pathway, which is termed autophagy flux, can be disturbed by bafilomycin A1, a specific inhibitor of vacuolar-type H+-ATPase. In the presence of bafilomycin A1, autophagosomes fail to exhibit the fusion with lysosomes, leading to the accumulation of numerous immature autophagosomes [18]. Thus, levels of the adaptor protein p62/SQSTM1 and the lipidated mature form of LC3 (LC3-II) increase in the presence of bafilomycin A1 and/or chloroquine under steady state or starvation conditions [19]. In this study, we chose to inhibit LC3B through miR-204 effect because miR-204 could target inhibit LC3B II directly to disturb autophagy-lysosome pathway.

The targeting effect of miR-204 on LC3B and its effect on chemoresistant of ovarian cancer cells has not been reported. Therefore, the mechanism of miR-204 in the vitro and vivo study which could through targeting LC3B to affect the response of ovarian cancer cells to chemotherapeutic drugs. The results showed that overexpression miR-204 could inhibit autophagy and promote apoptosis in ovarian cancer cells.

The results in ovarian cancer model of BALB/c nude mice showed the level of autophagy was significantly decreased and apoptosis was increased in the miR-204 agomir treatment group. It indicated that miR-204 could promote apoptosis by inhibiting autophagy, then affect the sensitivity of cancer cells to cisplatin. A Sacconi et al. found that down-regulation of miR-204 increased the expression of Bcl-2 in gastric cancer, while high expression of Bcl-2 reduced tumor response to 5-FU [20, 21]. Upregulation of miR-204 increases the chemosensitivity of gastric cancer cells to 5-FU and oxaliplatin through targeted inhibition of Bcl-2 [22]. When miR-204 level was decreased, tumor cells autophagy was increased, and autophagy could provide nutrients tumor cells to promote their proliferation and resistance [23, 24]. Conversely, increasing the level of miR-204 could effectively inhibit autophagy and promote tumor cell apoptosis. Above all, it indicated that miR-204 could play a pro-apoptotic role through inhibit LC3B and it could be a key miRNA to affect the way of tumor on programmed cell death. miR-204 targeting LC3B affects the sensitivity of ovarian cancer cells to cisplatin.

In order to further identify the clinical value of LC3B, we analyzed the data of ovarian cancer sample from TCGA and GEO databases. The results showed that high levels of LC3B had a worse prognosis. In multiple cancer such as squamous cell lung cancer, colon cancer and esophageal cancer, low levels of LC3B had a better prognosis. Analysis of pancancer data in TCGA also showed low level of LC3B had a better prognosis. These results suggest that LC3B may be a potential molecular marker for predicting the prognosis of tumors.

## Conclusion

In summary, it could be enhanced the sensitivity of ovarian cancer cells to chemotherapy by inhibited LC3B expression, and it was closely related to the level of miR-204. High levels of LC3B mean a poor prognosis. miR-204 could directly target LC3B to inhibit autophagy and promote apoptosis, thus enhancing the sensitivity of cancer cells to chemotherapy. The interaction between miR-204 and LC3B could influence the chemoresistant of ovarian cancer, and LC3B may be used as a biomarker to determine prognosis.

## Additional files

**Additional file 1: Table S1.** Summary of Ovarian cancer Patient’s Clinical and Pathologic Features. **Table S2.** Samples of EOC ascites and tissues. **Table S3.** Sequences of primers used for quantitative real-time-PCR. **Table S4.** List of primary antibodies used in western blotting. **Table S5.** Dates of luciferase reporter assay.

**Additional file 2: Figure S1.** Group diagram of ovarian cancer sample.

**Additional file 3: Figure S2.** The vitro study of LC3B in ovarian caner. (A) Cell viability assay of cisplatin-induced cytotoxicity in ovarian cancer cells. A2780, HO8910, SKOV3, and OVCAR3 cell viability were assessed by CCK-8 at 24 h after treatment with cisplatin. (B) Spearman correlation analysis of LC3B and MDR1 in TCGA and GEO data. (C) Expression of candidate miRNAs targeting LC3B in EOC. The expression levels of miRNAs were determined by qRT-PCR. Expression levels of miRNAs were compared between the sensitive and resistant groups in EOC samples, included 46 tumor tissues, 52 ascites cells. Data is presented as the mean ± SD. *p < 0.05, **p < 0.01, ***p < 0.001. (D) Comparison of miRNA expression levels between four ovarian cancer cells, included OVCAR3, SKOV3, A2780 and H8910. Data are presented as the mean ± SD. All assays were performed in triplicate and values represent the mean of three independent experiments. (E) Statistical analysis of tumor ROI values in BALB/c nude mice. (F) Spearman correlation analysis of LC3B and miR-204 in EOC samples, included 46 tumor tissues, 52 ascites cells.

**Additional file 4: Figure S3.** Cell viability assay and western blotting statistical analysis. (A) (B) Cell viability assay. Cell viability was assessed by CCK-8 after treatment of A2780 and OVCAR3 cells with cisplatin alone or in combination with miR-204 inhibitor/mimic or miR-negative transfection. *p < 0.05, **p < 0.01, ***p < 0.001. (C) Western blotting statistical analysis of cleaved-caspase3, caspase6 and caspase9 in A2780 cells. *p < 0.05, **p < 0.01, ***p < 0.001. (D) Western blots statistical analysis of cleaved-caspase3, caspase6 and caspase9 in OVCAR3 cells. *p < 0.05, **p < 0.01, ***p < 0.001. (E) (F) Western blotting statistical analysis of p-Akt and p-Bad in A2780 and OVCAR3 cells. *p < 0.05, **p < 0.01, ***p < 0.001. (G) qRT-PCR assay. Transfected with the miR-204 mimic increased caspase3 and caspase9 mRNA expression level in OVCAR3 cells. *p < 0.05, **p < 0.01, ***p < 0.001. (H) Transfected with the miR-204 inhibitor decreased caspase3 and caspase9 mRNA expression level in A2780 cells. **p < 0.01, ***p < 0.001. (I) Prognostic analysis of ESCA, COAD and LUSC tumor samples from TCGA data use GEPIA. High group > median LC3B, low group < median LC3B.

## Data Availability

All data generated or analysed during this study are included in this published article and its additional information files.

## Abbreviations

3′-UTRs: 3′-untranslated regions
3MA: 3-methyladenine
EOC: epithelial ovarian cancer
HRP: horseradish peroxidase
miRNA: microRNA
mRNA: messenger RNA
LC3B: microtubule-associated proteins 1A/1B light chain 3B
OS: overall survival
PFS: progression free survival
qRT-PCR: quantitative RT-PCR
PFA: paraformaldehyde
P-gp: P glycoprotein
TEM: transmission electron microscopy
SD: standard deviation
