# Supplementary material for: Inhibition LC3B can increase chemosensitivity of ovarian cancer cells
Source: Cancer Cell Int. 2019 Jul 29;19:199. doi: 10.1186/s12935-019-0921-z (PMC6664537; doi:10.1186/s12935-019-0921-z)
Supplement: Supplementary file 1 — Additional file 1: Table S1. Summary of Ovarian cancer Patient’s Clinical and Pathologic Features. Table S2. Samples of EOC ascites and tissues. Table S3. Sequences of primers used for quantitative real-time-PCR. Table S4. List of primary antibodies used in western blotting. Table S5. Dates of luciferase reporter assay. [file 12935_2019_921_MOESM1_ESM.doc]

**Table S1. Summary of Ovarian cancer Patient’s** Clinical and Pathologic Features

|  | Ovarian cancer ascites  n=116(%) | Ovarian cancer tissues  n=60（%） |
| --- | --- | --- |
| Age |  |  |
| ≤50 | 37(33.7) | 16(26.7) |
| >50 | 79(66.2) | 44(73.3) |
| Chemotherapy |  |  |
| No chemotherapy | 64(55.2) | 14(23.3) |
| Chemotherapy | 52(44.8) | 46(76.6) |
| Histological type |  |  |
| Serous | 116(100) | 60(100) |
| Stage |  |  |
| Ⅰ-Ⅱ | 19(16.4) | 11(18.3) |
| Ⅲ-Ⅳ | 97(83.6) | 49(81.7) |
| PFS |  |  |
| ≤6 months | 22/52(42.3) | 20/46(43.5) |
| >6 months | 30/52(57.7) | 26/46(56.5) |
| IHC of Ovarian cancer tissues |  |  |
| CA125 |  |  |
| - |  | 5(8.3) |
| + |  | 8(13.3) |
| ++ |  | 19(31.7) |
| +++ |  | 28(46.7) |
| MDR1 |  |  |
| - |  | 32(53.3) |
| + |  | 28(46.7) |

Table S2. Samples of EOC ascites and tissues

|  | EOC ascites (n=116) | EOC tissues (n=60) |
| --- | --- | --- |
| No chemotherapy group | 60 | 14 |
| Chemosensitivity group | 30 | 26 |
| Chemoresistance group | 22 | 20 |

**Table S3. Sequences of primers used for quantitative real-time-PCR**

| **Gene name** | **Primer Sequences** |
| --- | --- |
| miR-182 | RT:GTCGTATCCAGTGCAGGGTCCGAGGTGCACTGGATACGACAGTGTGA  Forward: TGCGGTTTGGCAATGGTAGAAC |
| miR-204 | RT:GTCGTATCCAGTGCAGGGTCCGAGGTATTCGCACTGGATACGACAGGCAT  Forward: TTCCCTTTGTCATCCTA |
| miR-211 | RT:GTCGTATCCAGTGCAGGGTCCGAGGTATTCGCACTGGATACGACAGGCGA  Forward: TTCCCTTTGTCATCCTT |
| miR-212 | RT:GTCGTATCCAGTGCAGGGTCCGAGGTATTCGCACTGGATACGACAGTAAG  Forward: ACCTTGGCTCTAGACTC |
| U6 | RT:GTCGTATCCAGTGCAGGGTCCGAGGTATTCGCACTGGATACGACAAAATATGGAAC  Forward: TGCGGGTGCTCGCTTCGGCAGC  Reverse: CCAGTGCAGGGTCCGAGGT |
| LC3B | Forward: GAGAAGCAGCTTCCTGTTCTGG  Reverse: GTGTCCGTTCACCAACAGGAAG |
| MDR1 | Forward: GGAGCCTACTTGGTGGCACATAA  Reverse: TGGCATAGTCAGGAGCAAATGAAC |
| Caspase3 | Forward: AGAACTGGACTGTGGCATTG  Reverse: CACAAAGCGACTGGATGAAC |
| Caspase9 | Forward: TTCCCAGGTTTTGTCTCCTG  Reverse: GGGACTGCAGGTCTTCAGAG |
| GAPDH | Forward: GGCCTCCAAGGAGTAAGACC  Reverse: AGGGGTCTACATGGCAACTG |
|  |  |

**TableS4**. List of primary antibodies used in western blotting.

| Antibody name | Company (Catalog No.) | Dilution |
| --- | --- | --- |
| Anti-Beclin1 | Abcam (ab62557 ) | 1：1000 |
| Anti-LC3B | Cell Signaling （3868S） | 1：1000 |
| Anti-Atg 7 | Cell Signaling （8558T） | 1：1000 |
| Anti-Caspase9 | Affinity biosciences（AF6348） | 1：1000 |
| Anti-MDR1 | Proteintech(22336-1-AP) | 1：500 |
| Anti-Cleaved-Caspase3 | Affinity biosciences（AF7022） | 1：500 |
| Anti-Caspase6 | Proteintech（10198-1-AP） | 1：500 |
| Anti-pAkt | Cell Signaling（4060S） | 1：1000 |
| Anti-pBad | Abcam(ab28824) | 1：500 |
| Anti-MMP2 | Proteintech(66366-1-Ig) | 1：1000 |
| Anti-MMP9 | Proteintech(10375-2-AP) | 1：1000 |
| Anti-P110 | Cell Signaling (3011S) | 1：1000 |
| Anti-GAPDH | Sungene biotech (KM9220T) | 1：5000 |
| Anti-Bax | Cell Signaling(2774S) | 1：1000 |
| Anti-Bak | Cell Signaling(12105S) | 1：1000 |
| Anti-Bcl-2 | Cell Signaling(15071S) | 1：1000 |
| Anti-cleaved-PARP | Cell Signaling(93790S) | 1：1000 |
| Anti-EpCAM | Cell Signaling(9272S) | 1：1000 |

**Table S5. Dates of luciferase reporter assay.**

| **Group** | **Result 1/Result 2**  （mean±SD） | ***p*** |
| --- | --- | --- |
| LC3B WT control group | 14.98±0.03 | *p*<0.0001 |
| LC3B WT miR-204 | 9.026±0.05 |
| LC3B MUT control group | 16.5±0.27 | *p*>0.05 |
| LC3B MUT miR-204 | 14.41±0.78 |
