# Supplementary figures and images for: Inhibition LC3B can increase chemosensitivity of ovarian cancer cells
Source: Cancer Cell Int. 2019 Jul 29;19:199. doi: 10.1186/s12935-019-0921-z (PMC6664537; doi:10.1186/s12935-019-0921-z)

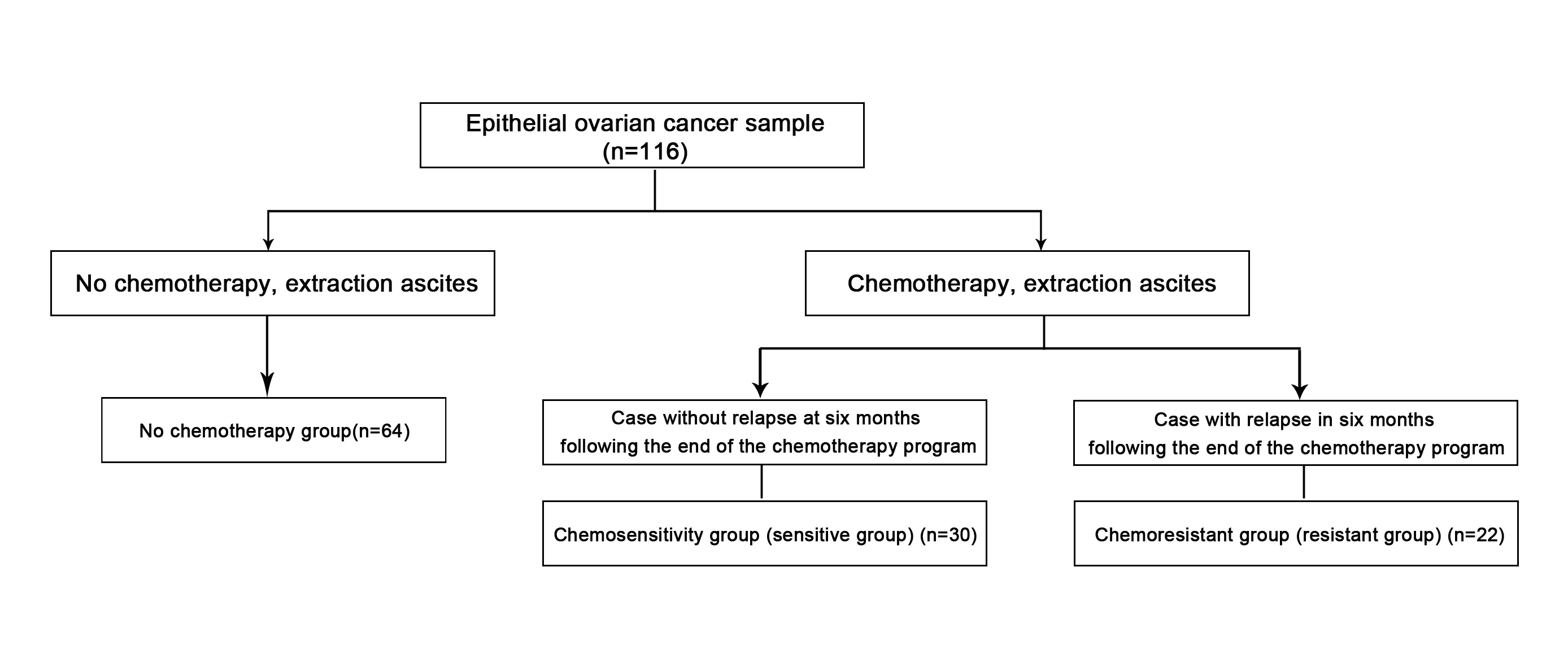

Supplement: Supplementary file 2 — Additional file 2: Figure S1. Group diagram of ovarian cancer sample. [file 12935_2019_921_MOESM2_ESM.jpg]

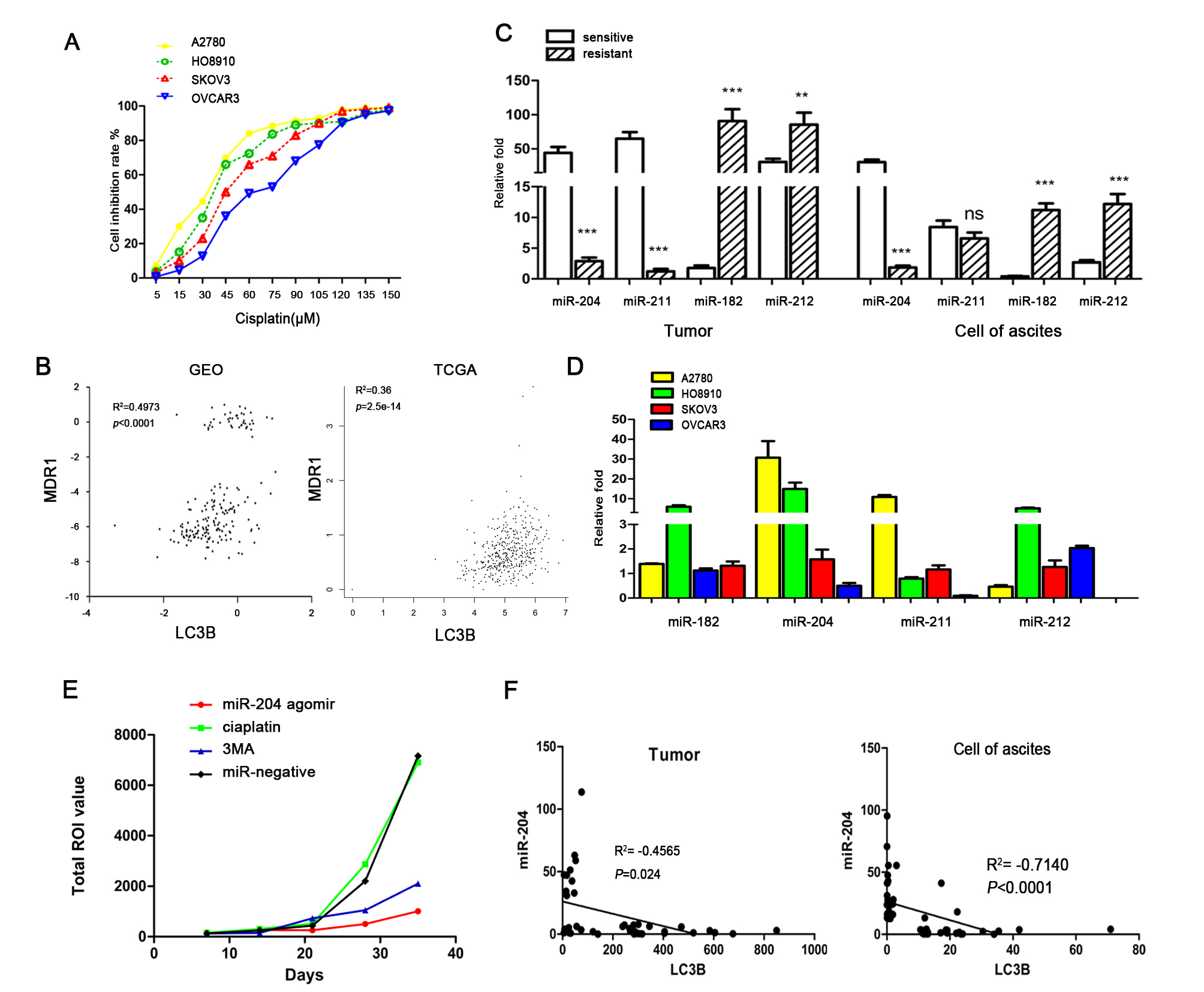

Supplement: Supplementary file 3 — Additional file 3: Figure S2. The vitro study of LC3B in ovarian caner. (A) Cell viability assay of cisplatin-induced cytotoxicity in ovarian cancer cells. A2780, HO8910, SKOV3, and OVCAR3 cell viability were assessed by CCK-8 at 24 h after treatment with cisplatin. (B) Spearman correlation analysis of LC3B and MDR1 in TCGA and GEO data. (C) Expression of candidate miRNAs targeting LC3B in EOC. The expression levels of miRNAs were determined by qRT-PCR. Expression levels of miRNAs were compared between the sensitive and resistant groups in EOC samples, included 46 tumor tissues, 52 ascites cells. Data is presented as the mean ± SD. *p < 0.05, **p < 0.01, ***p < 0.001. (D) Comparison of miRNA expression levels between four ovarian cancer cells, included OVCAR3, SKOV3, A2780 and H8910. Data are presented as the mean ± SD. All assays were performed in triplicate and values represent the mean of three independent experiments. (E) Statistical analysis of tumor ROI values in BALB/c nude mice. (F) Spearman correlation analysis of LC3B and miR-204 in EOC samples, included 46 tumor tissues, 52 ascites cells. [file 12935_2019_921_MOESM3_ESM.jpg]

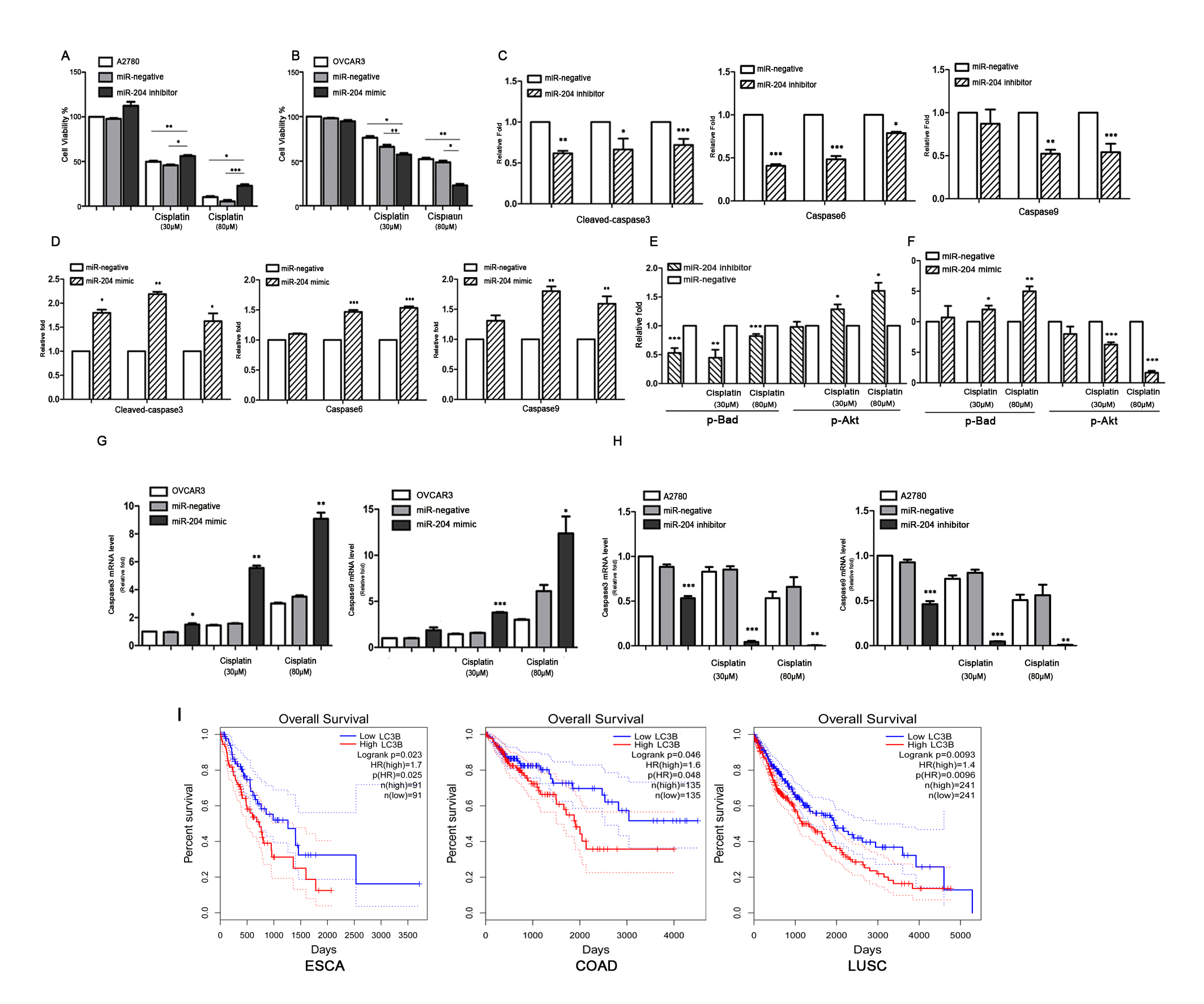

Supplement: Supplementary file 4 — Additional file 4: Figure S3. Cell viability assay and western blotting statistical analysis. (A) (B) Cell viability assay. Cell viability was assessed by CCK-8 after treatment of A2780 and OVCAR3 cells with cisplatin alone or in combination with miR-204 inhibitor/mimic or miR-negative transfection. *p < 0.05, **p < 0.01, ***p < 0.001. (C) Western blotting statistical analysis of cleaved-caspase3, caspase6 and caspase9 in A2780 cells. *p < 0.05, **p < 0.01, ***p < 0.001. (D) Western blots statistical analysis of cleaved-caspase3, caspase6 and caspase9 in OVCAR3 cells. *p < 0.05, **p < 0.01, ***p < 0.001. (E) (F) Western blotting statistical analysis of p-Akt and p-Bad in A2780 and OVCAR3 cells. *p < 0.05, **p < 0.01, ***p < 0.001. (G) qRT-PCR assay. Transfected with the miR-204 mimic increased caspase3 and caspase9 mRNA expression level in OVCAR3 cells. *p < 0.05, **p < 0.01, ***p < 0.001. (H) Transfected with the miR-204 inhibitor decreased caspase3 and caspase9 mRNA expression level in A2780 cells. **p < 0.01, ***p < 0.001. (I) Prognostic analysis of ESCA, COAD and LUSC tumor samples from TCGA data use GEPIA. High group > median LC3B, low group < median LC3B. [file 12935_2019_921_MOESM4_ESM.jpg]
